# Versatile Roles of LKB1 Kinase Signaling in Neural Development and Homeostasis

**DOI:** 10.3389/fnmol.2018.00354

**Published:** 2018-10-02

**Authors:** Ken-ichiro Kuwako, Hideyuki Okano

**Affiliations:** Department of Physiology, Keio University School of Medicine, Tokyo, Japan

**Keywords:** LKB1, kinase signaling pathway, neuronal development, neuronal homeostasis, neurite development

## Abstract

Kinase signaling pathways orchestrate a majority of cellular structures and functions across species. Liver kinase B1 (LKB1, also known as STK11 or Par-4) is a ubiquitously expressed master serine/threonine kinase that plays crucial roles in numerous cellular events, such as polarity control, proliferation, differentiation and energy homeostasis, in many types of cells by activating downstream kinases of the AMP-activated protein kinase (AMPK) subfamily members. In contrast to the accumulating evidence for LKB1 functions in nonneuronal tissues, its functions in the nervous system have been relatively less understood until recently. In the brain, LKB1 initially emerged as a principal regulator of axon/dendrite polarity in forebrain neurons. Thereafter, recent investigations have rapidly uncovered diverse and essential functions of LKB1 in the developing and mature nervous system, such as migration, neurite morphogenesis, myelination and the maintenance of neural integrity, demonstrating that LKB1 is also a multifunctional master kinase in the nervous system. In this review article, we summarize the expanding knowledge about the functional aspects of LKB1 signaling in neural development and homeostasis.

## Introduction

Liver kinase B1 (LKB1; Par-4 in *Caenorhabditis elegans* (*C. elegans*)) was originally identified by screening for genes that regulate the anterior-posterior axis in the *C. elegans* zygote (Kemphues et al., 1998). The human *LKB1* gene is widely expressed in various embryonic and adult tissues (Alessi et al., 2006; Katajisto et al., 2007; Jansen et al., 2009). The human LKB1 protein comprises 433 residues and consists of the N-terminal noncatalytic domain, the two nuclear localization signals, the kinase domain and the C-terminal regulatory domain. LKB1 possesses many conserved phosphorylation sites that are mainly enriched in the C-terminal regulatory domain and are highly conserved in *Drosophila*, *Xenopus* and mammalian LKB1 (Alessi et al., 2006). These phosphorylation sites are targeted by autophosphorylation (Thr185, Thr189, Thr336 and Ser404) or by upstream kinases (Ser31, Ser325, Thr366 and Ser431), such as cyclic AMP-dependent protein kinase A (PKA) and p90 ribosomal S6 protein kinase (p90RSK; Collins et al., 2000; Sapkota et al., 2002). Among the conserved phosphorylation sites, PKA- or p90RSK-mediated phosphorylation at Ser431 is particularly important for many LKB1 functions, such as epithelial polarity formation, cell-cycle control and axon specification (Sapkota et al., 2001; Martin and St Johnston, 2003; Barnes et al., 2007; Shelly et al., 2007). Upon activation, LKB1 in turn directly activates 14 downstream effector kinases of the AMP-activated protein kinase (AMPK) subfamily, including AMPKα1/α2, SAD-A/B, MARK1–4, SIK1–3, NUAK1/2 and SNRK, through the phosphorylation of a conserved threonine in the T-loop of the kinase domain (Lizcano et al., 2004; Jaleel et al., 2005). The activation of LKB1 is allosterically controlled by two cofactors, the LKB1-binding pseudokinase STE20-related adaptor (STRAD) and the scaffolding protein MO25. STRAD directly binds to LKB1, and this interaction is facilitated and stabilized by MO25, which binds to the C-terminal of STRAD (Baas et al., 2003; Boudeau et al., 2003). Once STRAD binds to LKB1, LKB1 is activated and translocated from the nucleus to the cytoplasm to exert its functions.

A number of studies on LKB1 have revealed that LKB1 serves as a master upstream kinase regulating various cellular processes such as cell polarity, cell-cycle control, gene expression and metabolic regulation in the whole body (Bright et al., 2009). In addition, the *LKB1* gene is mutated in Peutz-Jeghers syndrome, an autosomal dominantly inherited gastrointestinal cancer predisposition disorder that is characterized by the development of gastrointestinal polyps and abnormalities of mucocutaneous pigmentation (Hemminki et al., 1998; Jenne et al., 1998; Alessi et al., 2006). Moreover, the *LKB1* gene is frequently mutated in tumors such as cervical and lung cancer and is therefore considered a critical tumor suppressor gene (Sanchez-Cespedes, 2007; Jansen et al., 2009; Wingo et al., 2009).

In recent years, studies on the roles of LKB1 in the nervous system have greatly progressed, and it has become evident that LKB1 performs highly diverse functions in both neural development and homeostasis. In this review article, we will introduce recent advances in elucidating the neural functions of LKB1.

## Functions of LKB1 in Neural Development

### Neurite Development

Neurons are highly polarized cells with two distinct cellular compartments, the axon and the dendrite, which acquire specific structural and functional properties that enable neurons to relay information in the neural network (Craig and Banker, 1994). Using cultured hippocampal neurons, a number of molecules have been identified to be involved in axon specification, which establishes axon/dendrite polarity (Arimura and Kaibuchi, 2007). Although signaling pathways that control axon specification are intermingled with each other, along with the glycogen synthase kinase 3β (GSK3β) pathway, the LKB1 pathway is one of the major signals that positively regulate axon specification (Arimura and Kaibuchi, 2007; Shelly and Poo, 2011). The two original studies analyzing *LKB1*-deficient mice have revealed the essential role of LKB1 in axon specification by demonstrating that hippocampal and cortical neurons lacking LKB1 fail to form axons *in vitro* and abolish axon formation in the cortex (Barnes et al., 2007; Shelly et al., 2007). Combined knockdown and overexpression experiments in cultured neurons suggest that STRAD, an LKB1-binding protein that stabilizes LKB1, is required for LKB1-mediated axon specification (Barnes et al., 2007; Shelly et al., 2007). Furthermore, the phosphorylation level of SAD kinases, downstream effectors of LKB1, is dramatically decreased in the *LKB1*-deficient cerebral cortex, and the downregulation of SAD-A and SAD-B rescues the phenotype of multiple axon formation that is induced by LKB1 overexpression *in vitro* (Barnes et al., 2007), suggesting that SAD kinases mediate LKB1 to execute axon specification. In support of these studies on STRAD and SAD, either STRADα/β or SAD-A/-B double-knockout (KO) mice lack axons in the cortex (Kishi et al., 2005; Veleva-Rotse et al., 2014). SAD kinases regulate the phosphorylation of microtubule-associated proteins, such as tau, leading to alteration in microtubule organization that is critical for axon specification (Mandell and Banker, 1996; Kishi et al., 2005). These facts provide strong evidence for the pivotal role of the LKB1/STRAD-SAD kinase axis in the establishment of axon/dendrite polarity. In addition, overexpression of another STE20 family kinase Stk25, which binds to STRADα and Golgi matrix protein 130 (GM130), rescues the axon specification defect and the Golgi apparatus fragmentation that are induced by LKB1 knockdown *in vitro*, suggesting that Stk25 is involved in LKB1-mediated axon specification presumably through the dispersion of the Golgi apparatus (Matsuki et al., 2010). The upstream mechanism of LKB1-dependent axon specification has also been intensely studied. During the nuclear export of LKB1, Ran-binding protein 1 releases LKB1 from the nuclear export complex including exportins to ensure the cytoplasmic level of LKB1, and this mechanism is essential to LKB1-dependent axon specification, which is partly mediated by the Stk25-GM130 pathway (Mencarelli et al., 2018). The brain-derived neurotrophic factor (BDNF)-induced elevation of cAMP locally activates LKB1 in one neurite before axon differentiation via PKA-dependent phosphorylation at Ser431 (Shelly et al., 2007). cAMP and cGMP signaling act antagonistically on axon specification; cAMP promotes axon initiation through the phosphorylation of LKB1 and GSK3β, whereas cGMP suppresses it (Shelly et al., 2010). In contrast to BDNF, Sema3A elevates cGMP but reduces cAMP, thereby inhibiting the PKA-dependent phosphorylation of both LKB1 and GSK3β (Shelly et al., 2011). Since the local application of Sema3A is sufficient to inhibit the accumulation of phosphorylated LKB1 in undifferentiated neurites (Shelly et al., 2011), Sema3A signaling may contribute to axon specification by negatively selecting future axons. More recently, metabotropic γ-aminobutyric acid (GABA)_B_ receptors have also been shown to act as upstream negative regulators of LKB1 signaling during axon specification (Bony et al., 2013). The activation of GABA_B_ receptors reduces the cAMP-dependent phosphorylation of LKB1 and rescues the axonal defect of cortical neurons *in vivo* that is induced by the overexpression of LKB1 (Bony et al., 2013). Collectively, the cAMP/PKA-dependent phosphorylation of LKB1 and its association with STRAD activate the LKB1-SAD kinase pathway, leading to axon specification.

Notably, however, LKB1 and SAD kinases are not involved in axon/dendrite polarity formation in subcortical neurons, such as brainstem and spinal cord neurons (Lilley et al., 2013). In addition, LKB1 and SAD in *C. elegans* independently regulate neuronal polarity as two distinct pathways (Kim et al., 2010). These facts suggest that the contribution of the LKB1-SAD axis to axon specification may vary in different neurons depending on the cellular context.

LKB1 also controls the later stage of axonal development in cortical neurons. The deletion of *LKB1* or *NUAK1* in the cortex after the establishment of polarity clearly demonstrates that the LKB1-NUAK1 pathway is essential to axonal growth and terminal branching in cortical neurons *in vivo* (Courchet et al., 2013). This pathway immobilizes axonal mitochondria specifically at the nascent presynaptic site, and this mitochondrial immobilization in turn leads to axon branching (Courchet et al., 2013). Another study provides *in vitro* evidence that AMPK mediates the LKB1-dependent axonal growth of cortical neurons through activating PGC-1α a mitochondrial master regulator, to promote mitochondrial biogenesis that supplies sufficient levels of energy for growth (Vaarmann et al., 2016). However, the phosphorylation level of AMPK is not affected by *LKB1* deletion in the P0 cerebral cortex (Barnes et al., 2007), suggesting that AMPK is not a major effector of LKB1 functions in the developing cortex *in vivo*. Similarly, AMPK does not mediate LKB1-dependent eye development in *Drosophila*; instead, other downstream kinases may act as effectors of LKB1 in this case (Amin et al., 2009). Furthermore, AMPKα- or AMPKβ1-deficient mice show normal development of the nervous system, including the axogenesis of cortical neurons (Dzamko et al., 2010; Williams et al., 2011). Thus, the LKB1-AMPK pathway may have little, if any, impact on the development of the nervous system *in vivo*. Although SAD kinases are also required for the axonal arborization of neurotrophin-3-dependent sensory neurons and for the structural and functional maturation of presynapses in the peripheral and central nervous systems, LKB1 is not involved in these events of SAD-dependent axonal development at the later stages (Lilley et al., 2013, 2014).

In addition to its roles in axonal development, LKB1 is also directly engaged in dendrite development. The first evidence for the involvement of LKB1 in dendrite development comes from DA9 motor neurons in *C. elegans*. The genetic approach clearly demonstrates that Par-4 (LKB1) promotes the dendrite outgrowth of DA9 neurons as a downstream mediator of the UNC6 (Netrin)-UNC40 (DCC) pathway (Teichmann and Shen, 2011), even though the molecular mechanism by which Par-4 controls dendrite growth in this context has yet to be elucidated. Additional evidence in mice supports the distinct roles of LKB1 in dendrite development. In adult-born hippocampal granule cells, LKB1 is essential to the polarized initiation and oriented extension of the primary dendrite toward the molecular layer in the dentate gyrus by regulating the highly polarized distribution of the Golgi apparatus normally located at the base of the primary dendrite (Huang et al., 2014). The STRAD-Stk25-GM130 complex may mediate this LKB1 function in the asymmetric Golgi positioning, which is necessary for the oriented dendrite development, via stabilization of a Golgi signaling complex (Rao et al., 2018). Furthermore, LKB1 signaling controls dendrite spacing in cerebellar Purkinje cells. For many types of developing neurons, a mechanism called “dendrite self-avoidance,” by which sibling dendrites from the same neuron avoid crossing each other and clumping, is fundamental to establishing complete and nonredundant receptive field coverage to efficiently assemble synaptic inputs (Zipursky and Grueber, 2013). The cell-surface complex Slit2/Robo2-mediated repulsion mechanism is required for dendrite self-avoidance in Purkinje cells (Gibson et al., 2014). Deletion of *LKB1* in developing Purkinje cells drastically reduces dendritic Robo2 levels and impairs dendrite self-avoidance, and this effect of LKB1 is mediated by its substrate kinases SIK1 and SIK2 (Kuwako and Okano, 2018). Thus, the LKB1-SIK axis is essential for self-avoidance in Purkinje cell dendrites by regulating the dendritic sorting of Robo2. Although LKB1-mediated receptor sorting has not been demonstrated in other types of neuronal cells, LKB1 in lung cells promotes the cellular trafficking of the angiogenic receptor neuropilin-1, which is also involved in neurite development and neural migration, from the endosome to the lysosome for degradation, leading to attenuation of tumor angiogenesis and growth (Okon et al., 2014). Therefore, it is conceivable that LKB1 signaling also regulates other neuronal events, such as axon guidance, through receptor sorting toward specific cellular compartments.

**Figure 1:**
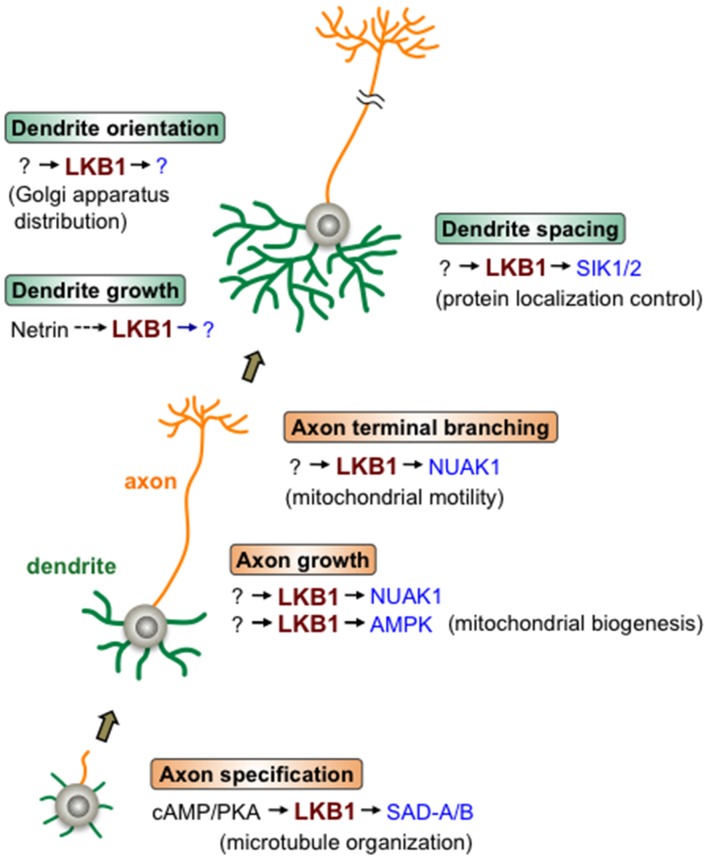
Liver kinase B1 (LKB1) signaling during neurite development. LKB1 signaling controls the various steps of axon/dendrite development. Beginning with axon specification, in which cAMP/protein kinase A (PKA) signal activates the LKB1-SAD-A/B pathway leading to microtubule organization, LKB1 controls axonal growth and branching as well as dendrite growth, orientation and spacing. LKB1 signaling regulates the cellular events described in the parentheses to accomplish each step of neurite development. The various downstream kinases (in blue) of LKB1, such as SAD-A/B, NUAK1 and SIK1/2, mediate different steps of neurite development. Note that in most cases, whether the depicted LKB1 functions are applicable to many types of neurons has not been evaluated.

In light of the combined evidence, LKB1 may sequentially function in a variety of steps in axon and dendrite development through adopting different downstream effector kinases, such as SAD-A/B, NUAK1 and SIK1/2, in the regulatory pathway of each event, highlighting the central roles of LKB1 signaling in the developmental program that constructs neurite architecture (Figure 1).

### Neuronal Migration

Neuronal migration is an essential process to establish the laminar architecture in the mammalian brain. In the developing cerebral cortex, young projection neurons undergo directional radial migration from the ventricular zone along the radial glial fibers toward the pial surface (Hatten, 1990). The cellular events during radial migration have been well defined; migrating neurons repeat highly organized sequential steps to reach their destinations (Tsai and Gleeson, 2005). First, migrating neurons form the leading process at the side of the pial surface by extending the leading edge along the radial glia fiber. Next, the centrosome, which is positioned at the apical side of the nucleus, is pulled up into the leading process by the force of the microtubule network. Finally, the nucleus translocates toward the centrosome, followed by the movement of the trailing cytoplasm to accomplish somal translocation. Hence, the correct positioning and movement of the centrosome is of great importance in neuronal migration. A pioneering study has shown that knockdown of LKB1 in immature cortical neurons disrupts the properly polarized positioning of the centrosome, thereby severely impairing neuronal migration (Asada et al., 2007). Subsequent research has clarified the molecular mechanism of LKB1-dependent neuronal migration in the developing cerebral cortex. LKB1 inactivates GSK3β through Ser9 phosphorylation at the tip of the leading process of migrating neurons (Asada and Sanada, 2010). This local inactivation of GSK3β leads to the association of adenomatous polyposis coli (APC), a microtubule-anchoring protein, with the distal end of the microtubule in the leading process tip, which stabilizes the microtubules (Zumbrunn et al., 2001). As a consequence, the APC binding-mediated microtubule stabilization enables the centrosomal forward movement and subsequent nuclear and somal translocations. Thus, LKB1-mediated microtubule stabilization in the tip of the leading process, via local inactivation of GSK3β followed by APC binding to microtubules, is a key mechanism for the migration of developing cortical neurons (Asada et al., 2007; Asada and Sanada, 2010). Other evidence supports the involvement of LKB1 in neuronal migration. STRADα knockdown impairs the migration of the developing cortical neurons (Orlova et al., 2010). Similarly, knockdown of Stk25, which regulates neuronal polarity by interacting with STRADα and GM130 (Matsuki et al., 2010; Rao et al., 2018), abrogates neuronal migration in the cerebral cortex (Matsuki et al., 2013). LKB1 also participates in the migration of neurons outside the cerebral cortex. In the developing cerebellum, differentiating granule cells migrate radially down from the external granular layer (GL) to the internal GL along the fibers of Bergmann glia. Specific deletion of *LKB1* in granule cell precursors results in the failure of their migration because of the decreased number of granule cells in the internal GL, which possibly leads to cortical expansion and the formation of extra lobes (Men et al., 2015a; Ryan et al., 2017).

### Myelination

Recent studies demonstrate that LKB1 regulates the Schwann cell-mediated myelination of peripheral axons through two different mechanisms. During postnatal differentiation, Schwann cells undergo drastic metabolic changes from glycolytic to mitochondrial oxidative metabolism, by which they produce the tricarboxylic acid cycle metabolite citrate, a precursor to cellular lipids. *LKB1*-deficient Schwann cells are unable to complete this metabolic shift, leading to insufficient synthesis of myelin lipids, thereby impairing the myelination of peripheral axons (Pooya et al., 2014). This finding suggests that LKB1-mediated metabolic reprogramming is essential to optimal myelination by Schwann cells. The LKB1-dependent activation of citrate synthase has been proposed to be a key mechanism of the metabolic shift in developing Schwann cells, even though the LKB1 downstream pathway involved in this machinery remains unknown (Pooya et al., 2014). In addition to this metabolic control, LKB1-mediated polarity control also contributes to myelination. The PKA-dependent phosphorylation of LKB1 leads to the asymmetric localization of LKB1 and Par-3, another polarity protein essential to the initiation of myelination, to the Schwann cell-peripheral axon interface (Shen et al., 2014). The specific deletion of *LKB1* in Schwann cells disrupts the asymmetric localization of Par-3 and results in hypomyelination (Shen et al., 2014). Thus, the PKA-LKB1 axis-dependent establishment of molecular polarity in the developing Schwann cells is required for the initiation and control of proper myelin extent. Although these two findings shed light on the importance of LKB1 in myelination of peripheral axons, whether LKB1 also regulates myelination by oligodendrocytes in the central nervous system has not been elucidated.

### Cell Polarity

LKB1 and its homologs control cell polarity in various types of cells across species, such as the *C. elegans* one-cell embryo, *Drosophila* oocyte and *human* intestinal epithelial cell (Watts et al., 2000; Martin and St Johnston, 2003; Baas et al., 2004), and LKB1 also plays key roles in establishing other types of cell polarity of neuronal cells in addition to axon/dendrite polarity. For example, LKB1 is required for the development and maintenance of cochlear hair cells in the inner ear. The proper alignment and orientation of two types of hair bundles, the stereociliary bundles and the transiently appearing kinocilium, which are located on the apical surface of hair cells and serve as mechanosensors, greatly contribute to the establishment of the planar cell polarity of hair cells (Rida and Chen, 2009). Deletion of *LKB1* in the developing hair cells causes the malformation of these hair bundles and the subsequent progressive death of outer hair cells, indicating that LKB1 signaling is essential for the planar polarity formation and survival of hair cells (Men et al., 2015b, 2016). In addition, *Drosophila* LKB1 regulates the asymmetric division of larval neuroblasts by controlling uneven cytokinesis as well as the proper localization of the protein complex Bazooka/atypical PKC/Par-6, that mediates the polarized distribution of the cell fate determinants Numb and Prospero, which are primarily sorted into a ganglion mother cell following cytokinesis, thereby establishing the identity of daughter cells (Bonaccorsi et al., 2007).

### Organelle Positioning in Diverse LKB1 Functions

Regulated organelle distribution is a key mechanism to construct highly polarized neural structures. As we mentioned above, LKB1 signaling-dependent regulation of the organelle distribution, including the Golgi apparatus, centrosome and mitochondria, mediates the various steps in neural development, such as axon specification, dendrite morphogenesis, axon branching and migration (Asada et al., 2007; Asada and Sanada, 2010; Matsuki et al., 2010; Courchet et al., 2013; Huang et al., 2014; Rao et al., 2018). Given that the LKB1-STRAD-Stk25 complex-mediated regulation of the Golgi dispersion is involved in both axon specification and dendrite development (Matsuki et al., 2010; Huang et al., 2014; Rao et al., 2018), same LKB1-dependent molecular mechanism for organelle positioning might be shared in distinct cellular events. Moreover, LKB1-mediated control of organelle positioning may commonly function in different types of neuronal cells to achieve a certain cellular event, such as neuronal migration that generally requires the regulated distribution of the centrosome (Solecki et al., 2006; Asada et al., 2007; Asada and Sanada, 2010). Thus, accumulating evidence indicates that the regulation of organelle distribution underlies the diverse LKB1 functions in the developing nervous system.

## Functions of LKB1 in the Mature Nervous System

Recent studies have also uncovered the roles of LKB1 in the mature nervous system. LKB1 signaling in hypothalamic proopiomelanocortin neurons regulates the peripheral glucose metabolism through the secretion of α-melanocyte-stimulating hormone (Claret et al., 2011). More recently, LKB1 was identified as a regulator of synaptic physiology. Mitochondria-dependent presynaptic Ca^2+^ homeostasis plays a key role in regulating neurotransmitter release (Billups and Forsythe, 2002). LKB1 controls the properties of neurotransmitter release at excitatory synapses of cortical neurons via regulation of the expression level of mitochondrial calcium uniporter that clears presynaptic Ca^2+^ (Kwon et al., 2016). These findings show that LKB1 is required for normal neural functions as well as neural development.

**Figure 2:**
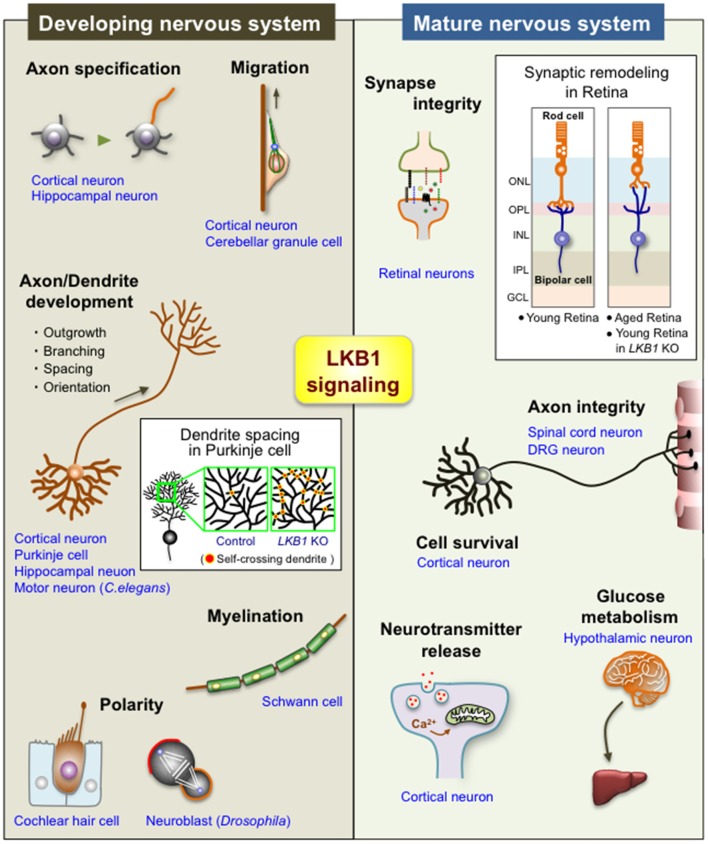
Diverse functions of LKB1 signaling in the developing and mature nervous system. The scheme shows the multiple functions of LKB1 in the developing and mature nervous systems. See the text for explanations of each function. The box in “Developing nervous system” shows a scheme of the dendrite spacing in Purkinje cells; there are many self-crossing dendrites in *LKB1* knockout (KO) Purkinje cells, whereas dendrites avoid self-crossing in control Purkinje cells via the mechanism of “dendrite self-avoidance,” indicating that LKB1 signaling is required for the proper dendrite spacing in Purkinje cells (Kuwako and Okano, 2018). The box in “Mature nervous system” shows a scheme of the synaptic remodeling of retinal neurons; the synapses between rod photoreceptor cells and bipolar cells (or horizontal cells (not depicted in the scheme)) are exclusively located on the OPL in the young retina, whereas those synapses are abnormally located in the ONL in the aged retina and in the *LKB1* KO young retina, indicating that LKB1 signaling is required for the maintenance of synaptic integrity in the retina (Samuel et al., 2014). ONL, outer nuclear layer; OPL, outer plexiform layer; INL, inner nuclear layer; IPL, inner plexiform layer; GCL, ganglion cell layer; DRG, dorsal root ganglion.

Several studies also demonstrate the crucial roles of LKB1 in maintaining the integrity of the nervous system. The LKB1-AMPK axis is involved in the synaptic aging of retinal neurons observed in old rodents and humans (Figure 2; Liets et al., 2006; Eliasieh et al., 2007; Terzibasi et al., 2009; Samuel et al., 2011, 2014). In the outer retina of young adults, rod photoreceptor cells form synapses with bipolar and horizontal cells exclusively in the outer plexiform layer (OPL; Figure 2). In the aged retina, however, the dendrites of horizontal and bipolar cells aberrantly extend far beyond the OPL into the outer nuclear layer (ONL) and form ectopic synapses with the retracted axons of rod cells. Age-related attenuation of the activity of the LKB1-AMPK pathway in rod cells leads to this synaptic remodeling in the outer retina of old mice (Samuel et al., 2014), suggesting that LKB1 signaling is essential to maintaining synaptic integrity (Figure 2). Importantly, increasing evidence supports that age-related synaptic dysfunction contributes to several neurodegenerative diseases, such as Alzheimer’s disease and Parkinson’s disease (Lepeta et al., 2016). Furthermore, abnormal regulation of AMPK has been implicated in those neurodegenerative diseases (Cai et al., 2012; Liu and Chern, 2015). Thus, the reduction in the LKB1-dependent activation of AMPK in the aged brain, which may in turn impair synaptic integrity, potentially underlies the pathogenesis of neurodegenerative diseases.

LKB1 is also involved in neuronal survival and axonal integrity. Mitochondrial dysfunction is a central mechanism implicated in numerous neurodegenerative diseases (Nunnari and Suomalainen, 2012). LKB1 plays a protective role against neuronal cell death following the loss of mitochondrial function by activating adaptive mechanisms that increase resistance to energy stress (Germain et al., 2013). Furthermore, *LKB1* deletion in the spinal cord concomitantly with the mid- and ventral brain causes axon degeneration in the thoracic spinal cord (Sun et al., 2011), indicating that LKB1 is indispensable for preserving axonal structure at least in a certain region of the nervous system. Glial LKB1 is also important in the mature nervous system. In addition to the pivotal role of LKB1 in the myelination of peripheral axons during development (Pooya et al., 2014; Shen et al., 2014), the LKB1 signaling-mediated energy control and lipid homeostasis in mature Schwann cells is essential to maintaining the integrity of sensory axons independently of myelination (Beirowski et al., 2014). In contrast to the beneficial functions of LKB1 in the differentiation and survival of developing hair cells (Men et al., 2015b, 2016), the LKB1-AMPK pathway mediates noise exposure-induced hair cell death and synaptopathy (Hill et al., 2016).

Of note, only AMPK has thus far been identified as a downstream effector of LKB1 in a few contexts in the mature nervous system. Thus, the detailed molecular mechanisms of the LKB-dependent control of neuronal homeostasis have yet to be unraveled.

## Concluding Remarks

To summarize the recent reports that collectively reveal the versatile neural functions of LKB1, LKB1 is now recognized as an essential kinase that supports many aspects of neural development and homeostasis (Figure 2). Nevertheless, there are several remaining issues regarding the roles and operating mechanism of LKB1 in the nervous system. First, given the rapid accumulation of evidence of novel neural functions of LKB1 in recent years, there must be further undiscovered LKB1 functions in the developing and mature brain. Second, the molecular mechanisms of LKB1-dependent controls in neural cells, particularly upstream signaling and downstream kinase(s), which activate and mediate LKB1, respectively, in each event, have not been well clarified in many cases. Lastly, whether the LKB1 functions previously identified in each context are universal for many types of neural cells remains unclear. Continued work exploring the yet-to-be-defined functions and molecular mechanisms of LKB1 signaling will facilitate understanding of the overall picture of LKB1-dependent programs that establish neural structures and functions.

## Author Contributions

KK and HO contributed to the writing and editing of the manuscript.

## Conflict of Interest Statement

The authors declare that the research was conducted in the absence of any commercial or financial relationships that could be construed as a potential conflict of interest.
